# The effect of empiric antimicrobial treatment duration on detection of bacterial DNA in sterile surgical specimens

**DOI:** 10.1371/journal.pone.0171074

**Published:** 2017-02-07

**Authors:** John Joseph Farrell, Huaping Wang, Rangarajan Sampath, Kristin S. Lowery, Robert A. Bonomo

**Affiliations:** 1 Department of Medicine, University of Illinois College of Medicine, Peoria, Illinois, United States of America; 2 Department of Laboratory Medicine, Division of Clinical Microbiology & Serology, OSF System Lab, Peoria, Illinois, United States of America; 3 Ibis Biosciences, an Abbott Company, Carlsbad, California, United States of America; 4 Research Service, Louis Stokes Cleveland Department of Veterans Affairs Medical Center, Cleveland, Ohio, United States of America; 5 Department of Medicine, Case Western Reserve University, Cleveland, Ohio, United States of America; 6 Department of Pharmacology, Case Western Reserve University, Cleveland, Ohio, United States of America; 7 Department of Molecular Biology and Microbiology, Case Western Reserve University, Cleveland, Ohio, United States of America; Seconda Universita degli Studi di Napoli, ITALY

## Abstract

Initial antimicrobial treatment of patients with deep seated or invasive infections is typically empiric. Usually, cultures of specimens obtained from the suspected source of infection are performed to identify pathogens and guide continued antimicrobial treatment. When patients present with signs and symptoms of infection, but sterile body fluid or tissue specimens cannot be obtained in a timely fashion, growth of bacterial pathogens in culture may be inhibited following initiation of empiric antibiotic treatment. To address this clinical dilemma, we performed a prospective evaluation of conventional culture vs. PCR coupled to electrospray ionization mass spectrometry (PCR/ESI-MS) on sterile body fluids and tissues submitted to the diagnostic microbiology lab following initiation of empiric antibiotic treatment for patients with suspected infection. In this series of surgical samples, PCR/ESI-MS identified bacterial pathogen(s) in 56% (49/87) of patients with non-diagnostic cultures. Examination of patients stratified by antibiotic treatment duration demonstrated that PCR/ESI-MS sustains high rates of bacterial DNA detection over time by generalized estimating equation models (p<0.0001).

## Introduction

Sepsis management guidelines recommend that blood cultures be obtained before the initiation of antibiotics. But even when blood culture collection precedes antibiotic treatment, blood cultures are negative in up to 50% of patients with severe sepsis or septic shock. [1, 2] Guidelines also encourage sampling of the suspected source of infection in a timely fashion, when the clinical circumstances allow. When the suspected source of infection cannot be readily sampled, several doses of empiric antibiotics may be administered prior to sampling or drainage, reducing the sensitivity for detection of bacteria in culture. [3]

Negative cultures of clinical specimens obtained from patients meeting systemic inflammatory response syndrome (SIRS) criteria for sepsis while receiving empiric antibiotic treatment may not result in discontinuation of the antibiotics. [2,4] When clinical criteria compel physicians to treat for infection despite negative cultures, reliance on institutional or community antibiograms to guide antimicrobial treatment typically results in use of overly broad empiric antibiotics. [4–6]

PCR coupled to electrospray-ionization mass spectrometry (PCR/ESI-MS) has demonstrated capacity for detection of bacterial DNA following initiation of antimicrobial treatment from serial culture negative clinical specimens obtained from patients with culture confirmed infection.[7] In a 2012 investigation, PCR/ESI-MS detected DNA evidence of bacterial pathogens in 60% of culture negative specimens obtained from inpatients following initiation of antibiotic treatment.[8] In this separate, prospective study we compare PCR/ESI-MS for detection of bacterial DNA vs. bacterial growth in conventional culture from sterile surgical specimens obtained from inpatients receiving antibiotic treatment. We also examined the relationship between duration of antimicrobial treatment and bacterial DNA detection by PCR/ESI-MS vs. growth in culture.

## Materials and methods

Following completion of a PCR/ESI-MS pilot study, approval was obtained from the University of Illinois College of Medicine and St. Francis Medical Center Institutional Review Boards for prospective PCR/ESI-MS testing of specimens submitted to the microbiology laboratory from inpatients receiving antimicrobial treatment at St. Francis Medical Center, Peoria, IL. All specimens were collected as part of the routine care of the patient and submitted to the microbiology laboratory by the treating physicians for conventional cultures. None of the patients included in this study were participates in the original pilot study.

Sterile specimens were collected during four one-week enrollments periods (July, 2013; October, 2013; March, 2014; and July, 2014) as opposed to one four week long enrollment period. During each one week enrollment period, inpatients who received at least one dose of oral or intravenous antibiotic treatment for suspected infection in the 24 hours prior to submission of sterile body fluids or tissues to the clinical microbiology lab for culture were eligible for inclusion in the study. Patient age, gender, vital signs at admission, suspected source of infection, number of empiric antibiotics received, white blood cell (WBC) count, days of antibiotic treatment (DOT) and number of antibiotics administered where recorded. Antibiotic treatments were classified recorded and classified into the following specific antibiotics or antibiotic classes: cephalosporins, fluoroquinolones, carbapenems, aminoglycosides, macrolides, piperacillin/tazobactam, vancomycin/daptomycin, penicillin/ampicillin/amoxicillin, tetracyclines, clindamycin, and antifungals. Patients receiving routine pre-operative prophylactic antibiotic treatment; immunocompromised patients, patients on chemotherapy for treatment of malignancy, or patients with HIV infection were excluded from this study (the research based PCR assay used in this study targets only bacteria and *Candida* spp.).

Sterile specimens included only specimens (either tissue or body fluid) collected in the operating room (OR) under sterile surgical conditions and placed in a sterile container, or body fluid obtained by percutaneous aspiration (PA) via syringe under appropriate aseptic technique; blood samples and blood cultures were not included in this study.

Gram stains, conventional aerobic and anaerobic culture and PCR/ESI-MS were performed on all specimens. Standard laboratory techniques during the study period included automated identification of organisms recovered in culture performed by the Vitek 2 instrument (bioMérieux, Durham, North Carolina). Once the specimen was processed by microbiology laboratory staff for Gram stain and cultures, the remaining specimen was categorized and grouped by source and placed in storage at 4°C while awaiting PCR/ESI-MS testing. The samples fell into eight source categories: abdominal, articular, bone, cardiovascular, head and neck, neurologic, soft tissue, and thoracic. Specimens were classified as thoracic, for example, if the sample came from within the chest but outside of the heart (*e*.*g*., pleural fluid), but as cardiovascular if the specimen was an intra-cardiac structure or device (*e*.*g*., heart valve tissue).

A research bacterial DNA detection assay (Abbott Molecular, Des Plaines, IL) was employed for nucleic acid amplification testing (NAAT) on a research PCR/ESI-MS platform (Ibis Biosciences, Carlsbad, CA). In each case, specimens were not frozen, but kept at 2–4°C until they were shipped overnight with a cold pack in an insulated shipping container to Ibis Biosciences (Carlsbad, CA). We followed the PCR/ESI-MS protocol previously established using a commercial test kit (BAC Detection Assay, Abbott Molecular). [9] Compared to clinical samples, the assay performs with 98.7% and 96.6% concordance at the genus and species levels, respectively [9].

Results were reported for all detections. Any growth in culture was considered a positive result with the exception of light growth of diphtheroids or coagulase negative *Staphylococcus* spp. For surgical cases, multiple specimens from the same site (or anatomically contiguous sites) were commonly collected in the OR and submitted together. For purposes of analysis, cultures were considered positive if growth was observed in any of the surgical specimens submitted from the OR.

We categorized patients into three antibiotic treatment groups, and examined the relationship between duration of antimicrobial treatment and bacterial detection by culture. Patients were stratified into three treatment groups based on days of antibiotic treatment (DOT): ≤ 2 days, 3–7 days, ≥ 8 days. The three strata were based on the two key clinical decision time points for hospitalized patients on empiric antibiotics with suspected infection: 2 days and 7 days (*i*.*e*., scrutiny of empiric antibiotic treatment for hospitalized patients with negative cultures is typically most intense at the 48 hr. and one week time points). [10] PCR/ESI-MS test results were not available to the patient or their treatment teams, and did not influence treatment decisions. [11] Pearson chi-square and Fisher’s exact tests were used to compare differences for all demographic and categorical variables (Table 1). Both the Kruskal-Wallis test and a general linear model were used to compare differences for continuous variables. Because both models produced very similar results, we reported *P* values from the general linear model with mean and standard deviations. Bonferroni correction was used for pairwise comparisons. We employed univariate analysis to examine the associations between clinical characteristics (*e*.*g*., site of infection, number of antibiotics, SIRS signs, specific antibiotic classes) and differences between PCR/ESI-MS and conventional culture results. Generalized estimating equations (GEE) were used to compare the difference between the two test methodologies (PCR/ESI-MS vs. Culture). Variables that were associated with a significant difference between PCR/ESI-MS and culture results in univariate analysis were adjusted for in multivariate analysis and included in the GEE models. The final GEE models only kept the significant covariates. All data analyses were conducted using SAS 9.4 (SAS Institute, Inc., Gary, NC). Two-tailed P values were calculated for all tests and P≤ 0.05 was the threshold for significance.

**Table 1:** Demographic, specimen source and patient treatment information.

Characteristics	Days of Antibiotic Treatment (DOT)			p value
	≤ 2 Days	3–7 days	≥ 8 Days	
	(n = 47)	(n = 43)	(n = 38)	
Age, Mean(SD)	55.4(23.2)	57.8(22.8)	51.1(19.1)	0.3893
Gender	No. (%)	No. (%)	No. (%)	
Men	24(51.1)	21(48.8)	20(52.6)	0.9424
Women	23(48.9)	22(51.2)	18(47.4)	
SIRS,	No. (%)	No. (%)	No. (%)	
0, 1	17(36.2)	14(32.6)	17(44.7)	0.5136
2,3,4	30(63.8)	29(67.4)	21(55.3)	
Empiric antibiotics	No. (%)	No. (%)	No. (%)	
Single therapy	17(36.2)	5(11.6)	4(10.5)	<0.001
Double therapy	24(51.1)	24(55.8)	13(34.2)	
≥ 3 antibiotics	6(12.8)	14(32.6)	21(55.3)	
PA vs. OR	No. (%)	No. (%)	No. (%)	
PA	26(55.3)	28(65.1)	18(47.4)	0.2714
OR	21(44.7)	15(34.9)	20(52.6)	
Specimen source	No. (%)	No. (%)	No. (%)	
Abdominal	10 (21.3)	5 (11.6)	6 (15.8)	
Articular	10 (21.3)	6 (14.0)	6 (15.8)	
Bone	2 (4.3)	1 (2.3)	1 (2.6)	
Cardiovascular	2 (4.3)	1 (2.3)	7 (18.4)	
Head & Neck	3 (6.4)	1 (2.3)	4 (10.5)	
Neurologic	3 (6.4)	5 (11.6)	3 (7.9)	
Soft tissue	5 (10.6)	2 (4.7)	3 (7.9)	
Valve	2 (4.3)	1 (2.3)	6 (15.8)	
Vascular	0 (0)	0 (0)	1 (2.6)	
Antibiotic treatment	No. (%)	No. (%)	No. (%)	p value
Vancomycin/Dapto	26(55.3)	29(67.4)	30(78.9)	0.071
Piperacillin/tazobactam	14(29.8)	20(46.5)	12(31.6)	0.2045
Cephalosporin	16(34)	17(39.5)	16(42.1)	0.733
Quinolone	8(17.0)	9(20.9)	10(26.3)	0.5794
Aminoglycoside	5(10.6)	5(11.6)	14(36.8)	0.003
Carbapenem	7(14.9)	9(20.9)	6(15.8)	0.7228
Penicillin/Amp/Amox	4(8.5)	1(2.3)	3(7.9)	0.3677
Macrolide	1(2.1)	4(9.3)	2(5.3)	0.3117
Tetracycline	1(2.1)	0(0)	3(7.9)	0.085
Clindamycin	3(6.4)	1(2.3)	4(10.5)	0.2916
Antifungal	1(2.1)	1(2.3)	1(2.6)	0.9885
WBC, Mean(SD)	13.5(7)	14.1(7.0)	13.4(5.9)	0.8752

Demographic, specimen source and treatment information stratified by Days of Antibiotic Treatment (DOT)

## Results

Our study population included 65 men and 63 women (51% and 49%, respectively). The average patient age was 55 years. Sterile specimens were obtained by PA for 56% (72/128) of patients. The mean and median DOT were 9 (S.D = 17.5) and 4 days, respectively with a range from 1–165 days. As expected, the treatment groups were slightly weighted towards DOT ≤ 2 days: 47 patients (37%) were treated for ≤ 2 days; 43 patients (33%) were treated for 3 to 7 days; and 38 patients (30%) were treated for ≥ 8 days. Sex and age were equally distributed for all three treatment groups, and no significant difference was observed among the three DOT groups in terms of sample source (PA vs. OR), number of SIRS present at admission, or WBC count. In contrast, patients with a DOT ≤ 2 days were much less likely to have received multiple empiric antibiotics than patients in the two longer DOT treatment groups (p<0.001). (Table 1)

The specimen samples most commonly submitted for culture following initiation of antibiotic treatment were: thoracic (42); articular (22); abdominal (21), neurologic (11), soft tissue (10), cardiovascular (10), head and neck (8), and bone (4).

The bacteria most commonly detected by either culture and/or PCR/ESI-MS were: *Staphylococcus aureus* (25); streptococci (23); and *Enterobacteriaceae* (10). Among culture negative patients, the DNA from bacteria most commonly identified by PCR/ESI-MS included: streptococci (16), *Enterobacteriaceae* (10), and *Fusobacterium nucleatum* (8). *S*. *aureus* was the most common organism detected by PCR/ESI-MS in culture positive specimens (18), with a trend towards decreasing recovery in culture at longer antibiotic treatment durations: DNA from *S*. *aureus* was detected by PCR/ESI-MS in 10/11 culture positive patients with DOT ≤ 2 days, but only 50% (3/6) of patients with *S*. *aureus* infection had growth in culture in the DOT ≥ 8 days cohort. In contrast, evidence of a similar decrease in detection by PCR/ESI-MS over time was not found.

PCR/ESI-MS detected DNA consistent with bacterial pathogens in 69.5% (89/128) of patients, but cultures were positive in only 32% (41/128) of patients. PCR/ESI-MS detections included 49/87 cases (56%) in which PCR/ESI-MS detected DNA from a bacterial pathogen in specimens from patients with non-diagnostic cultures (Table 2).

**Table 2:** Adjusted and unadjusted odds ratio of pathogen detection by DOT.

			Conventional Culture			
			Growth	No growth	Unadjusted	Adjusted
			n = 41	n = 87	OR(95% CI), *P* value	OR(95% CI), *P* value
Overall	PCR/ESI-MS	Detection	40	49	4.8(3.2,7.2),<0.0001	6.3(3.9,10.3); <0.0001
		No detection	1	38		
≤ 2 days	PCR/ESI-MS	Detection	25	4	1.3(0.9,1.9),0.1725	1.4(0.9,2.1); 0.1756
		No detection	1	17		
3–7 days	PCR/ESI-MS	Detection	9	24	12.5(5.3,29.6),<0.0001	24.2(6.8,86.2); <0.0001
		No detection	0	10		
≥ 8 days		Detection	6	21	13.1(5.0,34.1),<0.0001	16.7(6.0,46.4); <0.0001
	PCR/ESI-MS	No detection	0	11		

OR of pathogen detection for conventional microbiology vs. PCR/ESI-MS test results stratified by DOT

**Fig 1:**
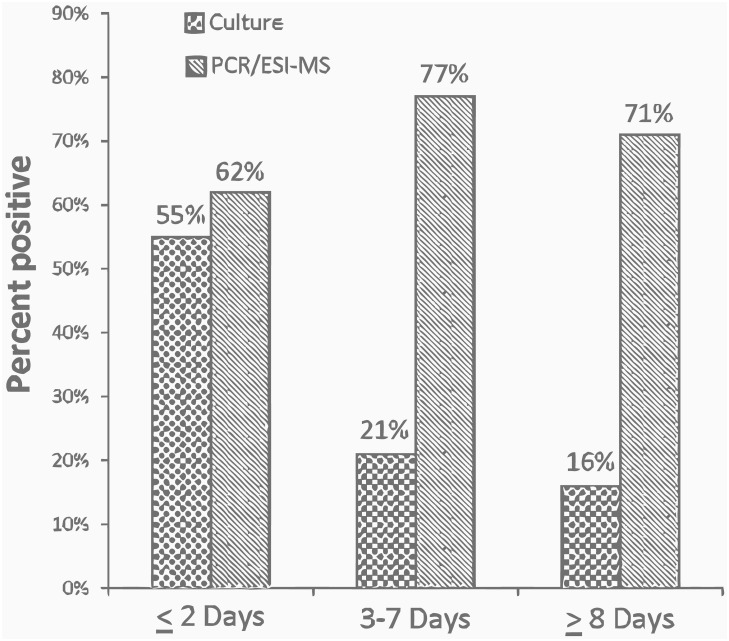
Culture vs. PCR/ESI-MS stratified by days of antibiotic treatment. Percent of a positive tests by culture vs. PCR/ESI-MS stratified by days of antibiotic treatment.

The total number of antibiotics administered as well as the percentage of positive cultures vs positive bacterial DNA detection by PCR-ESI-MS was compared across the three DOT strata. Exposure to ≥ 3 antibiotics was significantly more common in patients whose diagnostic sampling was performed ≥ 8 days following initiation of antibiotics (Table 1; column 5). For patients treated for ≤ 2 days, culture was positive for 55% (26/47) of patients, compared with 62% (29/47) for PCR/EIS-MS. For patients with a DOT of 3 to 7 days, only 21% (9/43) of patients were culture positive vs. 77% (33/43) of patients tested by PCR/ESI-MS. Likewise, among patients in the longest antibiotic treatment group (*i*.*e*., DOT ≥ 8 days) culture was only positive in 16% (6/38) of patients, compared with 71% (27/38) when the samples were tested by PCR/ESI-MS (Fig 1).

**Fig 2:**
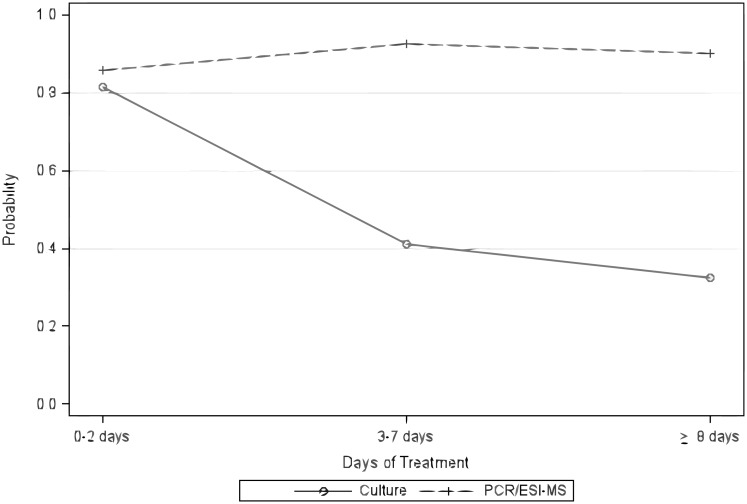
Probability of pathogen detection over time—culture vs. PCR/ESI-MS. Predicted probability of pathogen detection for culture vs. PCR/ESI-MS for sterile specimens obtained at DOT≤2, DOT 3–7 and DOT ≥8.

The number of SIRS criteria, number of empiric antibiotics, DOT, site of infection, and treatment with any of the following antibiotics: Vancomycin/Daptomycin, Piperacillin/tazobactam, or a cephalosporin, were associated with a significant difference between PCR/ESI-MS and culture results and were adjusted for in our multivariate analysis. We used GEE models to compare culture vs. PCR/ESI-MS by DOT after adjusting for the above variables.[11] A difference in the rate of pathogen recognition between culture and PCR/ESI-MS was not seen for DOT ≤ 2, but PCR/ESI-MS was significantly more likely than culture to detect DNA from a bacterial pathogen for DOT 3–7 (OR 24.2; 95% CI 6.8–86.2, p<0.0001), and DOT ≥ 8 (OR 16.7; 95% CI: 6.0–46.4, p<0.0001 (Table 2). The interaction plots of culture vs. PCR/ESI-MS by DOT and specimen source (OR vs. PA) illustrate the disparity between culture and PCR/ESI-MS for DOT 3–7 days, and DOT ≥ 8 days (Fig 2). The interaction plots also demonstrate a slightly higher likelihood of pathogen detection by PCR/ESI-MS in the longer treatment groups suggesting these strata are more likely to include patients with true infection than the DOT ≤ 2 group.

## Discussion

In our prospective investigation, PCR/ESI-MS was significantly more likely to detect bacteria than culture in sterile surgical specimens and body fluids obtained from patients who had received more than 2 days of antibiotic treatment (p<0.0001). PCR/ESI-MS also appeared useful for detection of bacterial pathogens at much longer antibiotic treatment durations. Predictive probability plots demonstrate a significant disparity between culture and PCR/ESI-MS for all patients with DOT > 2, with sustained high probability of pathogen detection for patients treated with antibiotics in both of the longer DOT groups (Fig 2).

Skepticism regarding the value of bacterial DNA detection from culture negative specimens is appropriate because negative cultures suggest that antimicrobial treatment is effectively suppressing the bacterial pathogens present in the sample, and negative cultures will not result in discontinuation of empiric antibiotic treatment when the treating clinician(s) remains suspicious of an infectious etiology. But data from this study show DNA detection of bacterial pathogens by PCR/ESI-MS in 56% of patients with negative cultures following initiation of antibiotics. If the pathogens detected by PCR/ESI-MS represent true false negative culture results this would have significant ramifications for clinicians in terms of choice and duration of antimicrobial treatment Identification of bacterial DNA by PCR/ESI-MS not only serves as validation of a recommendation for antimicrobial treatment when cultures are negative, but (along with identification of genetic markers of resistance) may provide alternative antimicrobial options to broad empiric antibiotic treatment. Most of the pathogens in this study detected by PCR/ESI-MS that were not recovered in culture (*e*.*g*., streptococci, *Enterobacteriaceae*, and *Fusobacterium*) are bacteria that could be treated effectively with a single antibiotic, but 32% (41/128) of all patients, and 55% (21/38) of patients with DOT ≥ 8, were treated with ≥ 3 antibiotics.

The premise of “salvage microbiology” is that species specific diagnostic information for patients with negative cultures from specimens collected following initiation of antimicrobial treatment may have value for clinicians and institutions striving to improve antimicrobial prescribing practices. There are many molecular platforms that offer both pathogen identification and genetic antimicrobial resistance detection, yet collaborative efforts between antimicrobial stewardship programs and microbiology laboratories to identify appropriate candidates for testing remains a novel idea. Prospective studies of the clinical and institutional value of salvage microbiology strategies are needed to confirm or refute our hypothesis that when sampling of suspected sites of infection is delayed, molecular methods of bacterial DNA detection such as PCR/ESI-MS for culture surgical specimens obtained from patients receiving empiric antibiotic treatment will result in decreased reliance on broad spectrum antimicrobial treatment.

## Supporting information

S1 File DE-identified treatment specimen and microbiology data. DE-identified study data with gender, WBC count, specimen source, treatment information and microbiology results.
(ODS)
